# Evaluation of Purdue Improved Crop Storage Triple Layer Hermetic Storage Bag against *Prostephanus truncatus* (Horn) (Coleoptera: Bostrichidae) and *Sitophilus zeamais* (Motsch.) (Coleoptera: Curculionidae)

**DOI:** 10.3390/insects10070204

**Published:** 2019-07-12

**Authors:** Kimondo Mutambuki, Hippolyte Affognon, Paddy Likhayo, Dieudonne Baributsa

**Affiliations:** 1 Kenya Agricultural and Livestock Research Organisation (KALRO), Food Crops Research Institute-Kabete, P.O. Box 14733-00800, Nairobi, Kenya; 2 West and Central Africa Council for Agricultural Development (CORAF), 7, Avenue Bourguiba-B.P. 48, CP 18523, Dakar, Senegal; 3 Purdue University, West Lafayette, IN 47907, USA

**Keywords:** PICS bags, *Prostephanus truncatus*, *Sitophilus zeamais*, grain storage, smallholder farmers

## Abstract

Hermetic technologies are being promoted in Africa as safer and more effective methods of grain storage on smallholder farms. However, farmers and policy makers lack knowledge of their efficacy in controlling major stored grain pests. An on-station study was conducted to evaluate the triple layer Purdue Improved Crop Storage (PICS) airtight bags against two major storage insect pests. Two sets each of PICS, jute and polypropylene bags were filled with 50 kg maize grain per bag. Each set was replicated four times. One set of PICS bags was each infested with 50 insects each of the larger grain borer *P. truncatus* and the maize weevil *S. zeamais*; while the other set was not. One set of jute and polypropylene woven bags was treated with a cocktail of 1.6% Pirimiphos methyl and 0.3% Permethrin, serving as positive controls; while the remaining sets with untreated maize grain formed negative controls. Gas analysis in the PICS bags followed the expected trend with oxygen levels falling sharply below 10% and carbon dioxide increasing to almost 10% after 12 weeks hence resulting in insect death. After 16 weeks, increase in oxygen levels may be attributed to perforation of the bags from outside by the *P. truncatus*. Results showed that PICS bags were significantly (*P* < 0.05) superior to treated and untreated controls of polypropylene and jute bags in suppressing insect development, maize grain damage and weight loss during storage. Weight loss in polypropylene and jute bags reached 40% and 41%, respectively, at 24 weeks after storage compared to PICS (2.4–2.9%). These results demonstrate that PICS bags can be used to store maize against *P. truncatus* and *S. zeamais* attack.

## 1. Introduction

Maize (*Zea mays* L.) is an important cereal grain grown widely in sub-Saharan Africa as a staple food crop [1,2] and contributes to food security of small-scale farmers [3]. While improved maize production practices have resulted in increased yields, poor on-farm postharvest handling and storage [4,5,6,7] have resulted in high grain losses. In Kenya, estimates of postharvest losses of maize caused by major storage insect pests vary from 24 to 48.5% [8,9,10,11,12,13] for untreated grains after six to nine months. The major storage insect pests include the larger grain borer *Prostephanus truncatus* (Horn) (Coleoptera: Bostrichidae), maize weevil *Sitophilus zeamais* (Motschulsky) (Coleoptera: Curculionidae) and angoumois grain moth *Sitotroga cereallela* (Lepidoptera: Gelechiidae) [7,14,15,16].

The control of stored product insect pests on smallholder farms remains a major challenge. Currently, synthetic insecticides are widely used to control insect pests of stored grains [17]. However, less than 25% of applied insecticides achieve good results leading to misperception of ineffectiveness [12]. Misuse (unplanned application, over-reliance on one active molecule and use of adulterated products) of insecticides [17] has resulted in insect resistance, chemical residues in grains and adverse effects on human and animal health [18]. In addition, heavy storage damage results in reduced quantity and nutritional content, sometimes rendering the grains unfit for human consumption, and low market value [19,20]. Readily available cost-effective storage devices that can reduce insect pest infestation and damage are therefore required.

Hermetic storage technologies such as the triple layer Purdue Improved Crop Storage (PICS), SuperGrain bags, AgroZ bags, GrainPro cocoons and others are being promoted as cheap and effective insecticide-free control devices against insect pests in developing countries [21,22,23,24,25]. Use of hermetically sealed containers to control major insect pests works by limiting oxygen access to insects, fungi and other microorganisms living inside the stored grain [26]. PICS bags have shown to be effective in controlling pests of several crops including cowpeas and maize [11,12,13,27,28]. Most of these studies have looked at the effect of a single insect pest on stored grain. This study was therefore set out to evaluate the efficacy of the PICS bags in protecting stored maize against the major occurring storage insect pests with special emphasis on *Prostephanus truncatus* and *Sitophilus zeamais* by simulating farmer storage conditions and practices, under ambient-conditions at the KALRO-Kiboko substation situated 160 km south east of Nairobi along the main Nairobi–Mombasa highway, an area with widespread of *P. truncatus*. The two insect pests co-exist in storage systems.

## 2. Materials and Methods

### 2.1. Study Site and Experiment Set-up

The evaluation was carried out at KALRO-Kiboko (37.7234°E and 2.2172°S) at 975 m above sea level in Makueni County. Kiboko is hot and dry, and is approximately 160 km along the Nairobi–Mombasa highway. The hottest months are February to March and September to October, before the onset of long and short rain seasons. The average and standard deviation of the daily maximum ambient temperature over the evaluation period was 31 ± 2 °C. The study was carried out during the long rain season under farmer-simulated conditions.

The experiment was established at the Kiboko KARLO sub-station in Kenya. 1200 kg of sieved white maize of hybrid 516 variety was divided into 24 bags (8 PICS bags, 8 jute bags and 8 polypropylene woven bags). The maize was harvested after the long rain season from experimental plots at KALRO-Kabete in Nairobi and kept in a store at ambient conditions for one and half months as data related to the experiments was obtained. This period conformed to farmer practices where maize is harvested, stored on cob to dry for 1–2 months before shelling and different protectants applied before storage. The maize was then purchased and transported to Kiboko for the present study. There were six treatments, consisting of:

(i) PICS + U: PICS bag + untreated maize, which simulated farmer practice of storing un-infested maize grain;
(ii) PICS + I: PICS bag + untreated maize + *Prostephanus truncatus* + *Sitophilus zeamais*; which simulated farmer practice of storing already infested maize grain with low levels of insect infestation;
(iii) PP+A: Polypropylene bag + maize treated with Actellic Super dust;
(iv) PP+U: Polypropylene bag + untreated maize;
(v) Jute+A: Jute bag + maize treated with Actellic Super dust;
(vi) Jute+U: Jute bag + untreated maize.

PICS bags were tested for air-tightness and found to be suitable. The 8 PICS bags were divided into two sets, one of which (4 replicates) was infested with 50 adult insects/bag of *P. truncatus* and *S. zeamais* (PICS + I) based on 1 insect per kilogram of grain for each species. Artificial low insect infestation was chosen to simulate residual insects in farmers storage systems. Hence each of the bags had in total 100 adult insects. No insects were introduced in the remaining set of the PICS bag (PICS + U). All PICS bags were securely tied by twisting and folding the lip of each layer with a strap to ensure air-tightness [29].

Two sets of the polypropylene (PP + A) and jute woven (Jute + A) bags were treated with calculated amounts (28 g) of actellic super dust (1.6% pirimiphos methyl and 0.3% permethrin) based on the recommended rate of 50 g per 90 kg grain bag. These served as positive controls. To ensure proper mixing of the insecticide dust with the grains, fuffle (a device that allows grain to pass through metal baffles thus achieving thorough mixing within a few seconds) was used in the admixing. The other two sets of 4 replicates each of polypropylene and jute bags were left untreated to serve as controls. All the bags were thereafter randomly distributed on wooden pallets (dunnage) in the experimental area inside the barn.

### 2.2. Insects

The two-week-old adult insects used in the study were obtained from the laboratory culture stocks at Kiboko. The *P. truncatus* (Coleoptera: Bostrichidae) and *S. zeamais* (Coleoptera: Curculionidae) were reared on whole susceptible hybrid maize at 27 °C and 65% relative humidity. To obtain the insects, a set of sieves (4.75 and 1.0 mm aperture size) were used to separate the grains and flour from the insects.

### 2.3. Data Collection

At the beginning of the experiment, a double tube compartmentalized spear was used to draw a representative sample of about 500 g from each bag and subsequently from same bag for grain quality analysis. Samples (500 g) from each bag were drawn by pushing the probe from top to bottom from four cardinal points after 8, 16 and 24 weeks of storage. Each sample was sieved to separate dust and free-living insects from grain. The weight of dust and the number of all live adult insects were recorded for each sample. Grain moisture content for each sample was measured using a pre-calibrated Dickey-John Multi-Grain Moisture Tester (Dickey-John Corporation, Illinois, USA). After measuring moisture content, each sample was then divided into four sub-samples of approximately 65 g; one of which was kept for reference. The three remaining sub-samples were each sorted out into insect-damaged and undamaged grain portions. Insect-damaged grains were those showing visible insect holes. The number and weights of each portion were recorded and the means of three sub-samples recorded for respective sample. Grain damage and weight loss, which is, a measure of loss caused by insect pests during the storage period was calculated according to count and weigh method [30], as follows:

Grain damage (%) = Number of insect-damaged grain × 100

Total number of grain.

$$\mathrm{Weight}\;\mathrm{loss}\;\left(\%\right)=\frac{\left(\mathrm{Wu}\times \mathrm{Nd}\right)-\left(\mathrm{Wd}\times \mathrm{Nu}\right)}{\mathrm{Wu}\times \left(\mathrm{Nu}+\mathrm{Nd}\right)}.$$

where W_u_ and W_d_ is the weight of undamaged and damaged grain, respectively; N_u_ and N_d_ is the number of undamaged and damaged grain, respectively.

Oxygen and carbon dioxide levels were measured using a MOCON PAC Check^®^ Model 325 Headspace analyzer (Mocon Inc, Minneapolis, MN, USA) at the onset of the experiment and every month until the end of the experiment. The MOCON^®^ gadget is fitted with a 20-gauge hypodermic needle for sampling gas inside the bag. To take the measurements, the outer and second layer of PICS bag were opened, and the inner liner pierced with the analyzer needle near the top, the tiny holes were then sealed with circular adhesive pads (10 mm diameter) immediately and reinforced with packing tape after taking the readings. Subsequent readings were taken from the spot by unsealing and resealing with the adhesive tape.

### 2.4. Statistical Analysis

The collected data were first organized in MS Excel to facilitate calculation of percentage grain damage and weight loss. Data were subjected to Shapiro-Wilk test for normality using the GenStat software Release 12.1 (VSN International Ltd 2009, Hemel Hempstead, UK). Where data did not show normal distribution, transformation to log_10_ (count + 1) for total live adult insect population and square root (√×) for percentage data (gas levels; moisture content level; grain damage and weight loss) were performed to normalize the variance. Bartlett’s test for homogeneity of variances was also performed. Data were first subjected to two-way analysis of variance (ANOVA) using the GenStat software. Treatment and storage time were the main effect. Gas levels, weight of dust, live insects, moisture content, grain damage and weight loss were the response variables. Afterwards and separately, one-way ANOVA of each variable was performed, with treatment as the main effect using General Linear Model procedure of GenStat and significant means were separated by Tukey HSD test at *P* < 0.05. However, for ease of understanding the untransformed means are presented.

Correlations between number of live insects and weight of flour, grain damage and weight loss were performed on transformed data using correlation procedure of Genstat at *P* < 0.05 level. The correlation between grain damage and weight loss was performed.

## 3. Results

### 3.1. Gas Composition Levels

**Figure 1:**
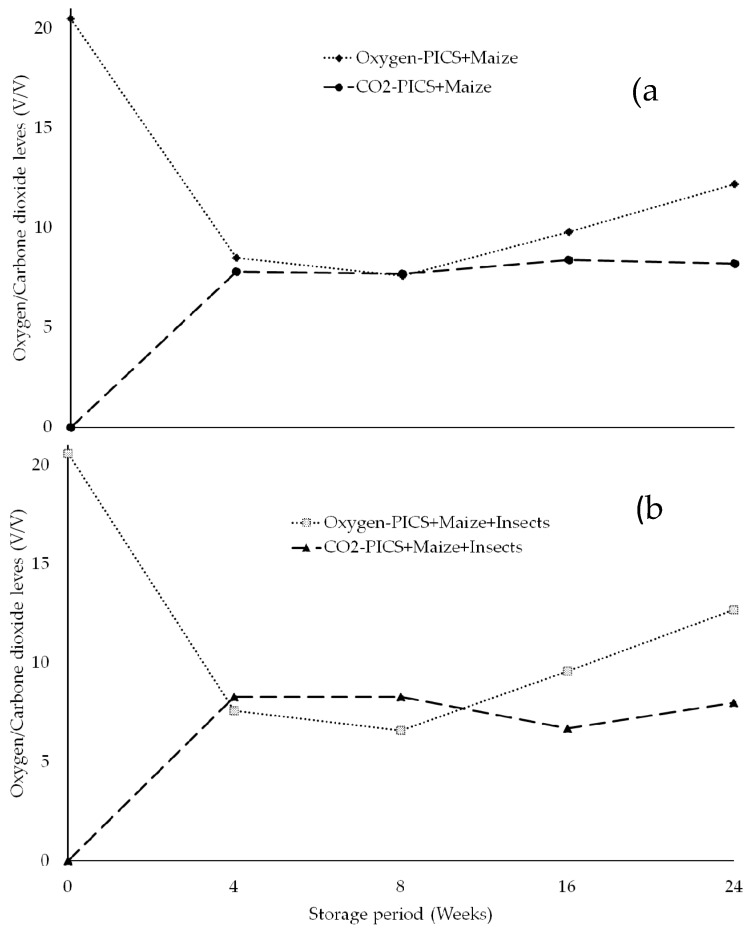
Percent levels of oxygen and carbon dioxide after 24 weeks of storage in (**a**) PICS bags filled with maize grain only and (**b**) PICS bags filled with maize grain and infested with insects (*P. truncatus* and *S. zeamais*).

The mean O_2_ and CO_2_ levels in PICS bags (infested and non-infested) are shown in Figure 1a,b. The levels followed the expected trend, with oxygen levels falling sharply from 20.5% to 8.5% and 7.6% for PICS non-infested and PICS infested, respectively, after four weeks of storage. On the other hand, carbon dioxide levels increased from 0.02% to 7.8% for PICS non-infested and 8.3% for PICS infested bags. This trend was maintained up to 16 weeks, whereupon the oxygen levels started rising.

### 3.2. Effect of Treatments on Grain Moisture Content Level

The mean grain moisture content at the start of the experiment was 12.6% (ranged from 12.4–13.0%). Grain moisture content varied significantly with treatment (F_5, 69_ = 5.77; *P* < 0.001) but not with storage time (F_3, 69_ = 2.52; *P* = 0.065). However, the interaction between the treatments and storage time was significant (F_15,69_ = 2.10; *P* = 0.02). The grains stored in PICS bags remained almost unchanged throughout the storage duration (Table 1). Although the moisture levels of grains stored in polypropylene and jute bags declined from the eight week, they were not significantly different from those observed in PICS bags at the end of storage time. There were no significant differences in the moisture levels of grains stored in polypropylene and jute bags over the entire storage duration.

### 3.3. Effect of Treatments on Weight of Dust

**Figure 2:**
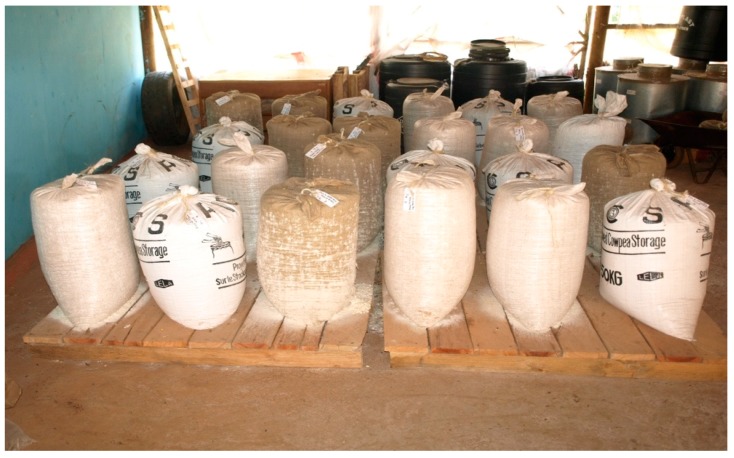
Side by side storage of PICS, PP and Jutes bags during the experiment at the KARLO research station in Kiboko, Kenya. Insects seriously damaged PP and Jute treatments, as reflected in the dust produced.

The initial average grain dust weight was 2.7 g and an upward trend in dust production was observed for all treatments except PICS bag (Table 2). The grain dust weight varied significantly with treatment (F_5, 69_ = 48.18; *P* < 0.001) and storage time (F_3,69_ = 100.58, *P* < 0.001). The interaction between the treatment and storage time was also significant (F_15,69_ = 24.39; *P* < 0.001). The amount of dust produced in a sample reflected insect feeding activity and hence the grain damage. PICS bag suppressed grain dust production compared to polypropylene and jute bags (Figure 2). Actual amount of dust after adjustment from the baseline was significantly low at only 0.3 g in PICS bag at the 24th week an indicator of low population of insects. The amount of dust in PICS bag over the storage period was practically not different (Table 2). Significant differences (*P* < 0.05) were only observed in untreated maize stored in jute bags (57 g) at 16 weeks. By the end of the trial, the untreated grain in polypropylene bags (100 g) and jute bags (117 g) showed significant differences to PICS and treated grain.

### 3.4. Effect of Treatments on Number of Live Insects

There were significant differences between the treatment (F_5,69_ = 59.16, *P* < 0.001) and storage time (F_3, 69_ = 361.41, *P* < 0.001). The interaction effect between treatments and storage time was significant (F_15, 69_ = 13.72; *P* < 0.001). Except for the baseline, on all sampling time point, significantly higher numbers of live adult insects were recorded in Actellic Super^®^ dust treated polypropylene and jute than PICS bags, while the same was observed in untreated grains from 16 weeks of storage (Table 3). The population of insects in grains stored in PICS bags with or without artificial infestation did not differ significantly. Up to the 16th week, only 1 to 2 adult *S. zeamais* but no *P. trunctus* insects were observed in the PICS bags. All *P. truncatus* in the sieved grain samples from these bags were dead. However, a drastic increase of live insects was evident at the end of the storage period (Table 3). Proliferation of insects in polypropylene and jute bags was higher than in PICS bags. Grains treated with Actellic Super^®^ dust did not suppress insect development, and although there were fewer than in untreated control grains, live adult insects were recorded in both polypropylene and jute bags throughout the storage period (Table 3).

**Figure 3:**
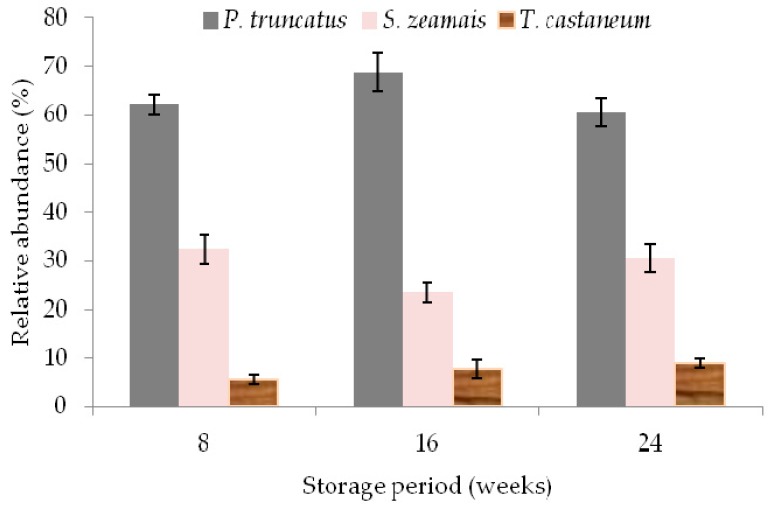
The percentage of insect species abundance across the treatments.

No significant difference of live insect infestation was found between treated maize in polypropylene and jute bags by the end of the trial. The percentage insect species abundance regardless of treatments is shown in Figure 3. In order of relative abundance, *P. truncatus* and *S. zeamais* were the most prevalent, followed by *Tribolium castaneum* (Herbst). Perforation of the two PICS liners from outside by *P. truncatus* started after the 16th week with several holes appearing at the end of the trial.

### 3.5. Effect of Treatment on Grain Damage and Weight Loss

The means of the percentage of insect-damaged grains are presented in Table 4. There were significant differences in percentages of insect-damaged grains with treatment (F_5, 69_ = 605.83; *P* < 0.001) and storage time (F_3, 69_ = 2432.94; *P* < 0.001). A high significant interaction effect of treatment and storage time on grain damage was detected (F_15,69_ = 208.35; *P* < 0.001). The baseline data showed that the maize had a low level of 5.6% insect-damaged grain (ranged from 4.8 to 6.8%) (Table 4). Marginal grain damage was detected in PICS bags with or without artificial infestation over the 24-week storage duration. Up to 8 weeks, there were no significant differences (*P* < 0.05) in the number of insect-damaged grains within the PICS bags PICS + U and PICS + I (Table 4). Differences, however, occurred at 16 weeks, but not at the end of the trial period. Between treatments, significant differences were observed in PICS and Actellic Super^®^ dust treated polypropylene and jute bags from 8th to 24th week of storage. In contrast, the percentage of insect-damaged grain in polypropylene and jute bags increased markedly, reaching 97.8% and 95.9%, respectively, after 24 weeks of storage. From the 8th week of storage, significantly higher proportions of insect-damaged grain were recorded in the polypropylene and jute bags compared to the PICS bags, a trend which was maintained over the entire storage duration. Unexpectedly, the percentage of insect-damaged grain in Actellic Super^®^ dust treated and untreated bags did not differ significantly, although higher levels of live adult insects were recorded at 24 weeks. Although the percentage damaged grain recorded in untreated jute bags was higher than that observed in polypropylene bags from the 16th to the 24th week, a significant difference was evident between the two treatments only at 16 weeks of storage. Perforation of the PICS bags from outside by *P. truncatus* towards the end of the trial is reflected by the slight increases in actual insect-damaged grain of 4 and 6% in PICS + U and PICS + I, respectively, compared to over 90% in the rest of the treatments, irrespective of the insecticide treatments used.

The means of grain weight loss are presented in Table 5. There were significant grain weight losses with treatment (F_5, 69_ = 3943.15; *P* < 0.001) and storage time (F_3, 69_ = 18127.06, *P* < 0.001). The interaction effect between treatment and storage time (F_15, 69_ = 1190.66; *P* < 0.001). Prior to the start of the trial, the average weight loss of the maize grain was 0.5% (ranged from 0.3% to 0.6%) (Table 5). Very low weight losses were observed in the PICS bags during the entire storage period. However, weight loss of grains stored in the polypropylene (PP + U) and jute (Jute + U) bags increased steadily to 40.6% and 41.5%, respectively, after 24 weeks of storage.

No significant differences were detected between grains stored in PICS bags with or without artificial infestation. From the 16th to 24th weeks of storage, significantly higher weight losses were recorded in both polypropylene and jute bags in comparison to PICS bags. There were no significant differences between the weight losses of grain stored in polypropylene and jute bags (PP + A, PP + U, Jute + A, Jute + U) at the end of 24 weeks of storage (Table 5).

Correlations between the number of live insects and weight of dust, grain damage and weight loss are presented in Table 6. Except for infested and uninfested PICS bags, high significant positive correlations were observed with weight of dust (Table 6). For all treatments, highly significant positive correlations were detected with grain damage and weight loss. Furthermore, there was a highly positive significant correlation between grain damage and weight loss (r = 0.992, *P* < 0.001).

## 4. Discussion

The gas composition in PICS bags showed that modified conditions due to aerobic respiration of the grain itself, insects and other microorganism was achieved. Although oxygen levels from 7.6 to 8.5% and from 7.8 to 8.3% carbon dioxide were recorded within four weeks of storage, extreme low levels of oxygen and high level of carbon dioxide were not attained. Despite low oxygen and increased carbon dioxide levels, the performance of PICS bags in suppressing insect development was not significantly affected. An oxygen level of 2–3% has been documented to make the feeding of insect larvae extremely slow or even stop thus resulting into death [26]. Lack of rapid depletion of oxygen and carbon dioxide build-up probably was due to low insect population. Minimum oxygen level was attained after eight weeks of storage, and thereafter a steady rise in the level towards normal was observed when the integrity of the liner was compromised by insect perforation from outside. The perforation of the liner allowed air to seep through into the surrounding airspaces between the grains in the bags hence may have supported insect activity. Previous studies on cassava and maize have shown limitations in retention of the modified conditions due to perforation of the PICS bag liners by storage insect pests [3,10,30]. Unexpectedly, carbon dioxide increased marginally in the perforated bags. This was probably due to a biological process that we cannot explain.

The study demonstrated that the impermeable PICS bag liner prevented changes in grain moisture content while that of grains stored in polypropylene and jute bags gradually change with storage period. Marginal grain moisture content variation was observed during storage in PICS bags. This could be attributed to the bags’ air-tightness which restricted humidity movement from outside to the inside of the bags in line with reports from previous studies [3,12]. The gradual decline in the moisture content of maize stored in polypropylene and jute bags can be explained by the change in prevailing environmental (hot and dry) conditions that characterize the study site. At the end of the storage period, significant increase in moisture content in untreated grains stored in jute bags was detected. The increase is attributed to very heavy insect infestation, collapse of permeable jute bag thus exposed grain to atmospheric conditions, localized hot spots and respiration that enabled the grains to absorb moisture. Similar observation in decline [12] and increase [11] in moisture content of maize stored in polypropylene bags after six months of storage have been documented.

Smallholder farmers store their maize grains to assure regular supply between harvest seasons. A decrease in live insect population was observed in PICS bag over the six-month storage period. This could be ascribed to the modified atmosphere created in the bags. However, once the integrity of air-tightness of the bags was lost (bags perforated by insects from outside), oxygen levels increased, enabling a slight increase in the live insect population 16 weeks after storage. Because all treatments (PICS, PP and jutes bags) were stored side by side (Figure 2), we believe that insects migrated from treated and untreated PP and Jute bags after seriously damaging the grain to the PICS bags. Treated and untreated PP and jute bags had high levels of grain damage, and insects might have left those bags in search of fresh maize. It is well documented that *P. trucantus* can bore into plastics, wood and other tough materials. Holes in PICS bags were made at the bottom of the bags, suggesting that *P. trucantus* bored into the wooden pallets (used it as traction) before making holes in PICS bags. Most of the live *P. truncatus* invading the bags from outside were congregated at the outer and inner layer where bags were tightly tied (knot). Further research on how to prevent perforation of the plastic liner by storage insect pests is required. However, one key lesson is not to store PICS bags along with heavily infested grain kept in PP or jute bags regardless of whether they are treated or not. The study results could probably have been different if tested on-farm, due to low insect population levels. The observed increase in insect population is consistent with previous studies [31,32]. A significant increase in live insect population occurred in polypropylene and jute bags, which was in accordance with earlier reports [10,13]. The widely used insecticide Actellic Super^®^ dust was ineffective against the storage insect pests 16 weeks after storage. This ineffectiveness can probably be attributed to excessive dust generated by the feeding activity of *P. truncatus* and other storage insect complex which diluted insecticide dust, thus explosing insects to a reduced contact amount of insecticide [32], and the loss of insecticidal potency of the active ingredients [33]. Similar observations have been documented in maize stored admixed with Actellic Super^®^ dust for six months in Kenya [34], Sofagrain™ dust (1.5% Pirimiphos-methyl + 0.5% Deltamethrin) in Ghana [35] and Chikwapuro^®^ dust (2.5% Pirimiphos-methyl + 0.1% Deltamethrin) in Zimbabwe [32].

The extent of insect infestation had an impact on the amount of dust generated by insect feeding, grain damage and weight losses. There was an increase in the amount of dust produced in polypropylene and jute bags as insect population increased. *Prostephanus truncatus* is known to produce copious amounts of dust during feeding (Figure 2). Hence the large amounts of dust in the controls (untreated woven and jute bags) compared to treated grain and grain held in the airtight PICS bag. Complementing the high dust levels was the maize weevil *S. zeamais*, as it bored through the kernels. The study demonstrated that correlation exists between the number of live insects and grain damage and weight loss for all treatments. However, no correlation was detected between weight of dust and number of live insects for PICS bags. This could be attributed to lower number of insects recorded in PICS bags compared to polypropylene and jute bags, which have been reported to be conducive for build-up insect population [12]. Furthermore, the correlation demonstrates that storing maize grain in PICS bags with or without insects does not affect its efficacy.

Grain damage which is evident by exit holes or tunneling made by adult or larval forms of insect influences the pricing of the grain at the market. A study in Ghana showed that maize grain damage levels higher than 5–6% attracted a 0.6–1.0% lower price for every 1.0% increase in grain damage [36]. Furthermore, a report on the extent that markets in Sub-Saharan Africa discount insect-damaged maize showed that the discount was 3% [37]. Although this study did not cover pricing of different grain damage levels, such information would be of great interest to smallholder farmers, thus motivating them to invest in hermetic storage devices. When highly infested, grain quality perception determines whether the grain could be used for human consumption or animal feed or destroyed [38,39]. The present study demonstrates the significant dust weight, grain damage and weight loss in maize stored in treated and untreated polypropylene and jute bags compared to that stored in PICS bags. Expectedly, the performance of treatments PICS + U and PICS + I were not significantly different, giving evidence that PICS bags offer good protection for both un-infested and low-infested grains. The high levels of dust weight, grain damage and weight loss measured in polypropylene and jute bags after 24 weeks may be explained by high rates of insect population. The extent of damage observed in polypropylene and jute bags with or without insecticide admixture after a storage period of 24 weeks rendered the grains unsuitable for human consumption, and thus would have been rejected on the market, hence resulting in an economic loss. The recorded extent of damage and losses were mainly caused by *P. truncatus* and *S. zeamais* infestation. However, expectedly, *Tribolium castaneum* (Herbst) was also recorded in polypropylene and jute bags after eight weeks of storage. The relative abundance of *T. castaneum* across the treatments was too low (<10%) to effect any significant grain damage or weight compared to *P.truncatus* and *S.zeamais*. While *T. castaneum* is a major insect pest of cocoa beans in both farm stores and warehouses [40], little is known about the extent of damage it causes on maize grain. Earlier studies show that *T. castaneum* was found in polypropylene and jute bags after initial infestation by *S. zeamais* [12], corroborating the observation of the present study. In PICS bags, reproduction of insects was suppressed due to the modified conditions of low oxygen and high carbon dioxide levels that prevailed. These modified conditions resulted in low grain damage and weight loss, which is in agreement with previous work [11]. Typically, the residual insect infestation under smallholder farmer conditions would be very low compared to a relatively high initial insect infestation density applied in the current study hence probably the obtained results would be different. To assess the actual performance of PICS bags in a normal farmer environment, it is suggested that the study be repeated in cluster of farmers’ homesteads under hot and dry, hot and humid, and cool and wet climatic conditions.

## 5. Conclusions

The findings show that PICS bags can prevent storage losses even in lowly infested maize grains without use of insecticide. Fairly modified atmosphere conditions were attained in PICS bags. The widely used Actellic Super^®^ dust was ineffective after 16 weeks of storage. Untreated maize grain stored in polypropylene and jute bags incurred high grain damage and weight losses. Farmers should not store PICS bags along with PP or jute bags containing highly infested maize.

## Figures and Tables

**Table 1:** Effect of treatment and storage period on percentage grain moisture content of maize stored for up to 24 weeks at the Kiboko KARLO research station in Kenya.

Treatments	Storage Period (Weeks)			
	0	8	16	24
PICS + U	12.5 ± 0.3a	12.7 ± 0.6a	12.6 ± 0.2bc	12.8 ± 0.5ab
PICS + I	12.9 ± 0.3a	13.0 ± 0.2a	12.9 ± 0.1c	13.3 ± 0.2b
PP + A	12.7 ± 0.2a	12.3 ± 0.1a	12.1 ± 0.2ab	12.1 ± 0.3ab
PP + U	12.5 ± 0.1a	12.6 ± 0.0a	12.6 ± 0.1bc	12.0 ± 0.2ab
Jute + A	13.0 ± 0.3a	12.8 ± 0.1a	12.2 ± 0.2ab	11.8 ± 0.1a
Jute + U	12.4 ± 0.4a	12.4 ±0.0a	11.7 ± 0.2a	12.6 ± 0.3ab
Mean	12.6	12.6	12.3	12.4
F_5, 15_-value	0.88	1.15	10.59	3.47
*P*-value	0.518	0.376	<0.001	<0.001

Means within the same column followed by the same letter are not significantly different at *P* = 0.05 level (Tukey test). PICS + U: PICS bag + untreated maize; PICS + I: PICS bag + maize + *Prostephanus truncatus* + *Sitophilus zeamais*; PP + A: Polypropylene bag + maize treated with Actellic Super dust; PP + U: Polypropylene bag + untreated maize; Jute + A: Jute bag + maize treated with Actellic Super dust; Jute + U: Jute bag + untreated maize.

**Table 2:** Effect of treatment and storage period on dust weight (grams) of maize stored for up to 24 weeks at the Kiboko KALRO research station in Kenya.

Treatments	Storage Period (Weeks)			
	0	8	16	24
PICS + U	2.6 ± 0.1a	2.7 ± 0.1a	2.9 ± 0.1a	2.9 ± 0.1a
PICS + I	2.5 ± 0.6a	3.0 ± 0.7a	3.1 ± 0.6a	3.4 ± 0.7a
PP + A	3.3 ± 0.1a	3.2 ± 0.5a	7.0 ± 1.6a	18.1 ± 1.4a
PP + U	3.3 ± 0.2a	3.5 ± 0.6a	9.5 ± 4.3a	100.0 ± 13.8b
Jute + A	2.3 ± 0.1a	2.5 ± 0.2a	6.0 ± 0.9a	33.9 ± 1.8a
Jute + U	2.4 ± 0.2a	3.7 ± 0.5a	57.2 ± 17.0b	116.9 ± 9.6b
Mean	2.7	3.1	14.3	45.9
F_5,15_-value	2.14	1.23	8.49	51.00
*P*-value	0.116	0.341	<0.001	<0.001

Means within the same column followed by the same letter are not significantly different at *P* = 0.05 level (Tukey test). PICS + U: PICS bag + untreated maize; PICS + I: PICS bag + maize + *Prostephanus truncatus* + *Sitophilus zeamais*; PP + A: Polypropylene bag + maize treated with Actellic Super dust; PP + U: Polypropylene bag + untreated maize; Jute + A: Jute bag + maize treated with Actellic Super dust; Jute + U: Jute bag + untreated maize.

**Table 3:** Effect of treatment and storage period on total number of live adult insects in maize stored for up to 24 weeks at the Kiboko KALRO research station in Kenya.

Treatments	Storage Period (Weeks)			
	0	8	16	24
PICS + U	1 ± 0a	0 ± 0a	2 ± 1a	15 ± 3a
PICS + I	0 ± 0a	1 ± 1ab	1 ± 1a	17 ± 5a
PP + A	0 ± 0a	9 ± 4c	22 ± 2b	171 ± 1b
PP + U	0 ± 0a	3 ± 1abc	50 ± 1c	545 ± 11d
Jute + A	0 ± 0a	7 ± 1c	43 ± 6b	136 ± 19b
Jute + U	0 ± 0a	5 ± 1bc	431 ± 3d	327 ± 2c
Mean	0	4	91	202
F_5,15_-value	2.25	8.93	30.00	42.88
*P*-value	0.103	<0.001	<0.01	<0.001

Means within the same column followed by the same letter are not significantly different at *P* = 0.05 level (Tukey test). PICS + U: PICS bag + untreated maize; PICS + I: PICS bag + maize + *Prostephanus truncatus* + *Sitophilus zeamais*; PP + A: Polypropylene bag + maize treated with Actellic Super^®^ dust; PP + U: Polypropylene bag + untreated maize; Jute + A: Jute bag + maize treated with Actellic Super^®^ dust t; Jute + U: Jute bag + untreated maize.

**Table 4:** Effect of treatment and storage period on percentage grain damage of maize stored for up to 24 weeks at the Kiboko KALRO research station in Kenya.

Treatments	Storage Period (Weeks)				
	**0**		**8**	16	24
PICS + U	4.8 ± 0.2a		6.0 ± 0.5a	4.3 ± 0.7a	8.8 ± 0.9a
PICS + I	6.0 ± 0.1a		7.7 ± 0.3ab	8.4 ± 0.4b	11.6 ± 1.3a
PP + A	5.4 ± 0.4a		8.9 ± 0.4b	4.9 ± 0.9c	93.8 ± 2.7b
PP + U	4.9 ± 0.4a		8.5 ± 0.1ab	40.7 ± 1.2d	95.9 ± 1.0b
Jute + A	5.8 ± 0.5a		9.2 ± 1.1b	38.0 ± 1.8d	92.6 ± 3.2b
Jute + U	6.8 ± 1.0a		7.9 ± 0.6ab	73.8 ± 1.8e	97.8 ± 0.4b
Mean	5.6	8.0	31.7	66.7	
F_5,15_-value	1.73	3.58	390.62	629.76	
*P*-value	0.187	0.025	<0.001	<0.001	

Means within the same column followed by the same letter are not significantly different at *P* = 0.05 level (Tukey test). PICS + U: PICS bag + untreated maize; PICS + I: PICS bag + maize + Prostephanus truncatus + Sitophilus zeamais; PP + A: Polypropylene bag + maize treated with Actellic Super dust; PP + U: Polypropylene bag + untreated maize; Jute + A: Jute bag + maize treated with Actellic Super dust; Jute + U: Jute bag + untreated maize.

**Table 5:** Effect of treatment and storage period on percentage weight loss of maize stored for up to 24 weeks at the Kiboko KALRO research station in Kenya.

Treatments	Storage Period (Weeks)			
	**0**	**8**	16	24
PICS + U	0.5 ± 0.2a	0.5 ± 0.0ab	0.6 ± 0.2a	2.4 ± 1.2a
PICS + I	0.3 ± 0.0a	0.4 ± 0.0a	1.1 ± 0.2a	2.9 ± 1.0a
PP + A	0.4 ± 0.1a	0.8 ± 0.1abc	11.4 ± 0.6b	39.7 ± 1.2b
PP + U	0.6 ± 0.2a	1.1 ± 0.2bc	16.6 ± 1.1c	40.6 ± 0.5b
Jute + A	0.5 ± 0.1a	1.6 ± 0.2c	15.4 ± 1.1bc	39.2 ± 1.4b
Jute + U	0.5 ± 0.1a	1.1 ± 0.3abc	29.5 ± 1.7d	41.5 ± 0.2b
Mean	0.5	0.9	12.4	27.7
F_5,15_ -value	1.64	42.13	76.20	11.27
P-value	0.209	<0.001	<0.001	<0.001

Means within the same column followed by the same letter are not significantly different at *P* = 0.05 level (Tukey test). PICS + U: PICS bag + untreated maize; PICS + I: PICS bag + maize + *Prostephanus truncatus* + *Sitophilus zeamais*; PP + A: Polypropylene bag + maize treated with Actellic Super dust; PP + U: Polypropylene bag + untreated maize; Jute + A: Jute bag + maize treated with Actellic Super dust; Jute + U: Jute bag + untreated maize.

**Table 6:** Correlations between: (i) live insects and weight of dust; (ii) live insects and weight of dust; and (iii) number of live insects and grain damage.

	Number of Live Insects and Weight of Dust		Number of Live Insects and Grain Damage		Number of Live Insects and Weight Loss	
Treatment	Correlation	*P*-Value	Correlation	*P*-Value	Correlation	*P*-Value
PICS + U	0.133	0.623	0.682	0.004	0.834	<0.001
PICS + I	0.317	0.231	0.700	0.002	0.854	<0.001
PP + A	0.855	<0.001	0.920	<0.001	0.922	<0.001
PP + U	0.861	<0.001	0.972	<0.001	0.961	<0.001
Jute + A	0.841	<0.001	0.917	<0.001	0.935	<0.001
Jute + U	0.971	<0.001	0.954	<0.001	0.963	<0.001

Where PICS + U: PICS bag + untreated maize; PICS + I: PICS bag + maize + *Prostephanus truncatus* + *Sitophilus zeamais*; PP + A: Polypropylene bag + maize treated with Actellic Super dust; PP + U: Polypropylene bag + untreated maize; Jute + A: Jute bag + maize treated with Actellic Super dust; Jute + U: Jute bag + untreated maize.
